# 0.045% DAC N-055 a choice for poor Afghan surgical patients?

**DOI:** 10.1186/1753-6561-5-S6-P246

**Published:** 2011-06-29

**Authors:** KW Stahl, H Darmangar

**Affiliations:** 1 Waisenmedizin E. V., Freiburg i. Brsg., Germany; 2 Medical Faculty, Balkh University, Mazar-e-Sharif, Afghanistan

## Introduction / objectives

O_2_ enriched pharmaceutical chlorite [NaClO_2_ German Drug Codex monograph DAC N-055, 1990] contains Na_2_Cl_2_O_6_ formerly called TCDO, (NaClO_2_)_4_·O_2_ fights tissue infections and promotes wound granulation of radiogenic ulcers [http://www-pub.iaea.org/mtcd/publications/pdf/te_1300_web.pdf]. Under our hygienic conditions the intra- and postoperative topical use of 0.045% DAC N-055 helps us to deal with bone & tissue infections in orthopaedic surgery in Mazar. Here we document 4 cases out of the total number of 26 patients, we monitored in the former Military Hospital in Mazar 2004 - 2006 to familiarize ourselves with this German drug first registered as Oxoferin^®^ in 1983.

## Methods

The speed of ^·^ClO_2_ release from 0.045 % DAC N-055 (max. 160 ppm) increases with decreasing pH. ^·^ClO_2_ induces no resistance. In all patients the field of surgical intervention is rinsed several times with 0.045 % DAC N-055 in saline especially during the operation, especially before wound closure and dressed with cotton gauzes kept moist till the patient is discharged home. Post-OP irrigations are practised with 0.045 % DAC N-055 for septic arthritis (closed method) and osteomylitis (open method).

## Results

1/2 septic arthritis, osteomyelitis cases and 2/22 osteomyelitis cases are photo-documented. Without antibiotics, septic arthritis had an excellent functional outcome after 6 weeks, the open fracture with skin defect received mesh graft on excellent granulation after 1 month and was completely closed after 3 months. 11/22 osteomyelitis cases could be monitored for 6 to 18 months, 6 cases relapsed within this time, for 8/11 patients antibiotics were not available.

## Conclusion

If 4.5% DAC N-055 could be sold in Afghanistan at 50% of the price of the finished drug Oxoferin^®^ sold in Pakistan this could help poor patients in traumatology.

## Disclosure of interest

None declared.

